# Antemortem diagnosis of asbestosis by screening chest radiograph correlated with postmortem histologic features of asbestosis: a study of 273 cases

**DOI:** 10.1186/1745-6673-4-14

**Published:** 2009-06-12

**Authors:** Kelly N Mizell, Christopher G Morris, J Elliot Carter

**Affiliations:** 1 University of South Alabama, Department of Pathology, 2451 Fillingim Street, Mobile, Alabama 36617, USA

## Abstract

**Background:**

Accuracy in the clinical diagnosis of asbestosis has significant implications for the future health of affected patients as well as serious medicolegal implications for both patients and asbestos-associated industries. The radiographic gold-standard for diagnosis of asbestosis has been the plain chest radiograph, and in many asbestosis-screening clinics, chest radiograph abnormalities in conjunction with a history of asbestos exposure have been the mainstay of diagnosis. No studies have yet compared the antemortem chest radiographic diagnosis of asbestosis with the subsequent presence of pulmonary fibrosis and lung tissue ferruginous bodies at autopsy.

**Methods:**

Records were reviewed from 273 autopsies performed to investigate asbestosis over an 11-year period. Accrued data included age and gender as well as the presence or absence of the following: occupational exposure to asbestos, antemortem clinical diagnosis of asbestosis by chest radiograph, fibrous pleural plaques, peribronchiolar or interstitial pulmonary fibrosis, ferruginous bodies in histologic sections of lung tissue, and ferruginous bodies in digested lung tissue.

**Results:**

242 cases with the antemortem radiographic diagnosis of asbestosis (study group) were identified. 31 additional autopsies had been requested based on history of asbestos exposure without radiographic documentation of asbestosis (control group). Comparison of the two groups showed a statistically significant increase in the association of chest radiograph-diagnosed asbestosis and the presence at autopsy of pleural plaques (p = 0.0109), peribronchiolar or interstitial pulmonary fibrosis (p = 0.0472), and histologically-diagnostic asbestosis (p = 0.0021). At autopsy, histologically-diagnostic asbestosis was confirmed in only 90 of the 243 study group cases. Comparison of individual parameters within the 242 study group cases showed a statistically significant correlation between the presence of fibrous pleural plaques and histologically-proven pulmonary fibrosis (p = 0.0025) as well as the subsequent histologic diagnosis of asbestosis (p < 0.0001).

**Conclusion:**

Clinical diagnosis of asbestosis by screening chest radiograph is more predictive of the postmortem presence of fibrous pleural plaques, pulmonary fibrosis, and histologically-proven asbestosis than is occupational exposure history alone. However, chest radiograph-based diagnosis of asbestosis significantly overpredicts the subsequent histologic diagnosis of asbestosis.

## Background

Asbestosis is a disease of interstitial pneumonitis and fibrosis caused by the inhalation of asbestos fibers [1]. The antemortem diagnosis of abestosis is typically made on clinical grounds without the aid of histologic evidence [2]. The American Thoracic Society (ATS) published a statement in 1986, which was revised in 2004, regarding the clinical diagnosis of asbestosis [3]. This statement concludes that there must be evidence of structural pathology of the lung documented by imaging or histology, evidence of causation by asbestos (by exposure history, by histologic demonstration of asbestos bodies, or by other means), an appropriate interval between exposure and disease, and exclusion of alternative causes for the findings [1]. For most patients, a history of exposure to asbestos and a chest radiograph demonstrating changes consistent with asbestosis have been used to meet these criteria [3]. Chest radiograph findings considered consistent with asbestosis include lower lung zone reticulonodular infiltrates and small irregular opacities, pleural thickening, and obliteration of the cardiac border [3]. Since the 1950s, the International Labor Office (ILO) classification scheme for pneumoconiosis has standardized the radiographic diagnosis of asbestosis [1]. This system, when combined with the "B-reader" qualification for persons considered competent to classify pneumoconiosis films, is intended to maintain consistency in classifying chest radiographs of patients with suspected pneumoconiosis [1]. Previous studies have evaluated the correlation between antemortem detection of pleural plaques on chest x-ray and postmortem findings of pleural plaques on histologic examination [4]. However, the histologic diagnosis of asbestosis is generally accepted as demonstration of peribronchial fibrosis and asbestos bodies in tissue sections [3] and does not include pleural plaques [1]. On review of the medical literature, no studies comparing the antemortem chest radiographic diagnosis of asbestosis with the subsequent presence of pulmonary fibrosis and lung tissue ferruginous bodies at autopsy could be identified. The goal of this study is to examine the relationship between antemortem diagnosis of asbestosis by chest radiography and postmortem presence of the accepted histologic criteria for diagnosis.

## Methods

Records of post-mortem examinations performed over an 11-year period (January 1990 – December 2001) were searched for autopsies conducted to investigate an antemortem diagnosis of asbestosis at the University of South Alabama Medical Center in Mobile, AL. This was accomplished by a systematic search of autopsy records and by requesting specific records on patients with asbestos exposure who had been referred by a regional law firm that used screening chest radiograph as their antemortem diagnostic standard. This search yielded 242 cases with an antemortem chest radiographic diagnosis of asbestosis which were used as the study group. An additional 31 cases were identified in which there was a history of asbestos exposure but no antemortem radiographic diagnosis of asbestosis. These 31 cases were used as a control group. For each of the 273 cases, the age and gender of the patient were recorded along with the presence or absence of the following: history of occupational exposure to asbestos, antemortem clinical diagnosis of asbestosis by chest radiograph using the ILO classification, fibrous pleural plaques, peribronchiolar pulmonary fibrosis, ferruginous bodies in histologic sections of lung tissue, and ferruginous bodies in digested lung tissue. For the purpose of statistical analysis, asbestosis was considered histologically-proven if both peribronchiolar fibrosis and multiple ferruginous bodies were present in histologic sections of lung tissue.

JMP software was used for statistical analysis to compare the presence of fibrous pleural plaques, peribronchiolar or interstitial pulmonary fibrosis, tissue and digestion ferruginous bodies, and histologically-proven asbestosis between the study group and control group (SAS Institute, Cary, NC). Data were examined by contingency table, and Fisher's exact test provided assessment of statistical significance.

## Results

Our search yielded 242 cases with an antemortem chest radiographic diagnosis of asbestosis (age range 38–91, mean = 70.7 years). An additional 31 cases were identified in which there was a history of asbestos exposure but no antemortem radiographic diagnosis of asbestosis (age range 42–86, mean = 70.2 years). Comparison of the two groups showed an increase in the association of chest radiograph-diagnosed asbestosis and the presence at autopsy of pleural plaques (61.1% in the CXR-positive group vs. 35.4% in the control group), peribronchiolar or interstitial pulmonary fibrosis (64.8% vs. 45.1%), tissue ferruginous bodies (41.3% vs. 12.9%), ferruginous bodies in digested lung tissue (75.5% vs. 46.4%), and histologically-diagnostic asbestosis (36.8% vs. 9.7%) (see Table 1). This association was found to be statistically significant for all of these pathologic findings (p = 0.0109 for pleural plaques, p = 0.0472 for periobronchiolar or interstitial pulmonary fibrosis, p = 0.0016 for tissue ferruginous bodies, p = 0.0028 for digestion ferruginous bodies, and p = 0.0021 for histologically-proven asbestosis). At autopsy, histologically-diagnostic asbestosis was confirmed in only 89 of the 243 study group cases. Comparison of individual parameters within the 242 study group cases showed a statistically-significant correlation between the presence of fibrous pleural plaques and histologically-proven pulmonary fibrosis (p = 0.0025) as well as the subsequent histologic diagnosis of asbestosis (p < 0.0001).

**Table 1:** Comparison of the antemortem chest radiograph diagnosed group vs. the group diagnosed by history alone

	Positive Chest Radiograph			Control Group			P-value
	Yes	No	% Yes	Yes	No	% Yes	
Pleural Plaques	148	94	61.1	11	20	35.4	0.0109

Fibrosis	157	85	64.8	14	17	45.1	0.0472

Tissue Ferruginous Bodies	100	142	41.3	4	27	12.9	0.0016

Digestion Ferruginous Bodies*	169	55	75.5	13	15	46.4	0.0028

Histologically proven	89	153	36.8	3	28	9.7	0.0021

* It should be noted that the total number of cases listed for ferruginous bodies tissue digestion does not equal 273 because there

were cases in both the study group and the control group in which tissue digestion was not performed or documented.

## Discussion

Chest radiographs in patients with asbestosis may show ground-glass opacification, small opacities, a blurred cardiac silhouette and poorly-defined diaphragmatic contours [5]. In more advanced disease, honeycombing and volume loss may be seen [5]. These changes are more pronounced in the lower-lobes, but can extend to involve the middle lobe, lingula and upper lobes in advanced cases [5].

Pleural plaques are the most common manifestation of asbestos exposure [5]. These areas of fibrosis usually arise from the parietal pleural, but may also arise from the visceral pleura and occur 20–30 years after exposure [5]. They are most often seen on the posteriolateral chest wall between ribs 7 and 10, on the lateral chest wall between ribs 6 and 9, on the dome of the diaphragm and on the mediastinal pleura [5]. Several previous studies have investigated the correlation between radiographic diagnosis of pleural plaques and their presence at autopsy [4]. These have shown that the percentage of pleural plaques present at autopsy that were detected by premortem chest radiography ranged from 8–40% and that the plaques may be found in up to 39% of the general population at autopsy [3]. Studies have also shown that pleural plaques, if present in a patient with exposure to asbestos, may indicate an increased risk of mesothelioma and laryngeal carcinoma, but are not a precursor lesion for either [3].

The results of this study indicate that a chest radiograph suggestive of asbestosis (using ILO standards) combined with a history of asbestos exposure is more predictive of histologically-proven asbestosis at autopsy than exposure history alone. All of the pathologic findings associated with asbestosis were found at a statistically-significant increased rate in the study group (those with history and chest radiographs consistent with asbestosis) compared to the control group (those with history of exposure to asbestos but without diagnostic chest radiography).

Previous reports have suggested that chest radiographs may underestimate the presence of histologically-proven asbestosis, particularly in the early stages of the disease [6,7]. In order for pulmonary fibrosis to produce irregular opacities on chest radiograph, there must be enough fibrotic change to produce a summative effect that allows it to become radiographically detectable [8]. It has been reported that 10–14% of patients in previous studies who had autopsy-proven asbestosis had normal antemortem chest radiography [6]. Another study of patients with lung cancer and asbestos exposure showed that although 100% of the patients had histologic evidence of parenchymal fibrosis, 18% had no radiographic evidence of parenchymal fibrosis, and 10% had radiographic evidence of neither parenchymal fibrosis nor pleural disease [8]. This follows a similar trend for patients with any diffuse infiltrative lung disease, of whom approximately 10% have been shown to have normal chest radiography [7]. The current study, however, indicates that chest-radiography over-predicts the histologic diagnosis of asbestosis by a wide margin. Previous studies have shown that, when films are read by radiologists provided with patient history indicating the possibility of an exposure, there is a tendency for over-reading [7]. As the majority of the patients in our study were referred by law firms, it is likely a safe assumption that the radiologists knew of a potential exposure to asbestos in the majority of the cases.

The Mobile, Alabama area has a large shipbuilding industry, and the majority of autopsy cases in our study were referred by a local law firm involved in legal action against these companies. Because of this industry in our area, there was a large population of patients with long-term and significant asbestos exposure. This may explain the high percentage of patients with known exposure who had histologic evidence of asbestosis regardless of radiographic evidence for the diagnosis (36.8 percent of patients with radiographic evidence and 9.7% of those without radiographic evidence).

**Figure 1:**
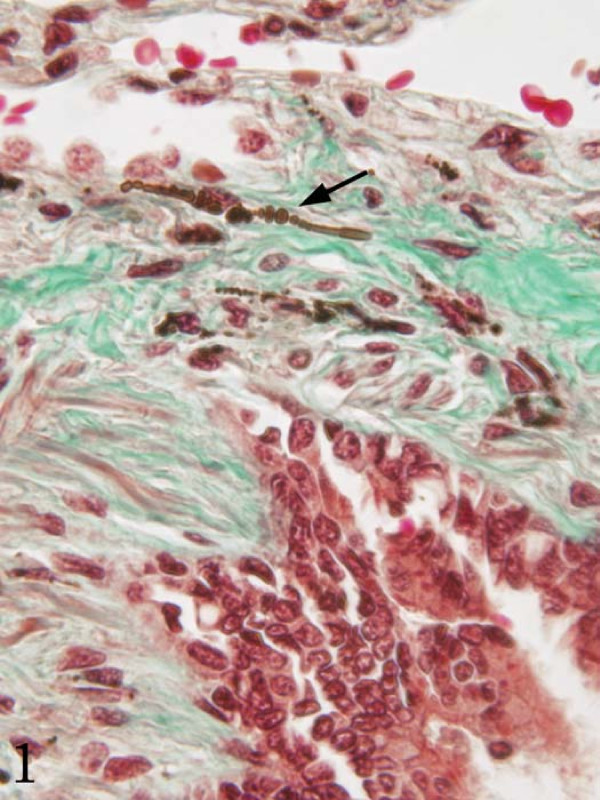
**Histologic asbestosis: tissue ferruginous body associated with peribronchiolar fibrosis (Masson trichrome stain, 40×)**.

**Figure 2:**
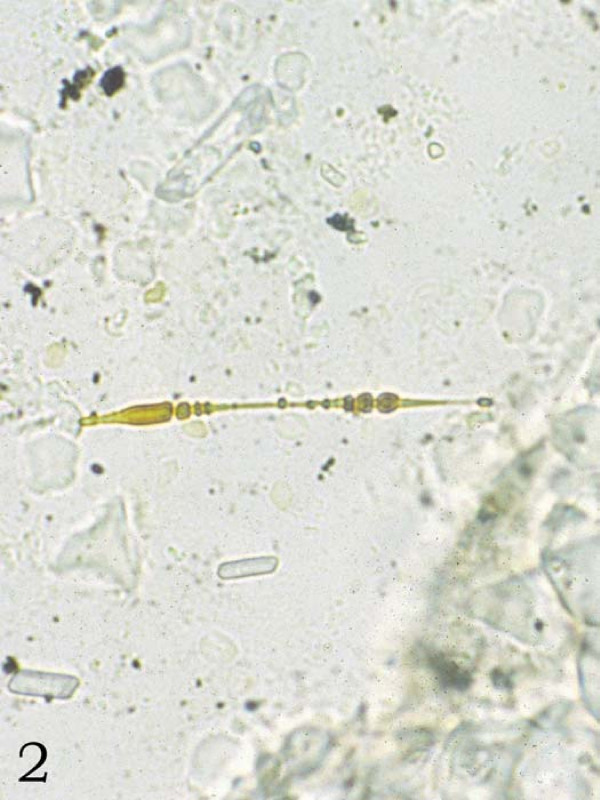
**Asbestos fiber identified by lung tissue digestion studies (unstained, 40×)**.

The histologic diagnosis of asbestosis is made when diffuse pulmonary interstitial fibrosis is found along with asbestos bodies in lung tissue (Figure 1) [3]. Asbestos bodies are golden-brown, fusiform rods with a translucent center that are made of asbestos fibers coated with an iron-containing material (Figure 2) [3]. Other inorganic particulates may become similarly coated, and if no asbestos core is seen, they are best known as ferruginous bodies [3]. Although much of the research into the correlation of radiographic evidence of asbestos-related changes and their findings at autopsy has focused on pleural changes (i.e. pleural plaques and pleural fibrosis), these findings are not part of the histologic criteria for asbestosis. However pleural plaques and pleural fibrosis may be an indicator of exposure to asbestos. It has been suggested that the incorporation of the asbestos-related pleural diseases under the heading of asbestosis should be avoided as this groups together diseases with different epidemiology and clinical outcomes [3].

Quantification of asbestos fibers in lung tissue digestion has historically produced widely variable results, even on the same sample in the same lab. This method involves chemical digestion of lung tissue with recovery and concentration of the mineral fibers. These fibers can then be analyzed by some form of microscopy. Light microscopy, phase contrast light microscopy, scanning electron microscopy and transmission electron microscopy have all been used for this purpose [3]. Most investigators have preferred transmission electron microscopy because it provides the highest resolution for the identification of the smallest fibers, and diffraction studies can be used which help differentiate the various types of fibers. Despite the variability, previous studies have shown that there is a correlation to increased fiber burden in tissue digestion with more severe fibrosis [3]. Although each lab would have to set its own normal range, Roggli et al. suggest that a patient with pulmonary interstitial fibrosis who has fewer than 10^6 ^fibers of 5 μm or greater in length per gram of dried lung (10^5 ^for wet lung) is unlikely to be suffering from asbestosis [3]. Our study found ferruginous bodies identified by lung tissue digestion were more common in patients with a radiographic diagnosis of asbestosis than those without (p = 0.0028). While previous studies have shown that at least 10^5 ^fibers are needed to be clinically significant, at our institution, digestion methods changed over the course of the review period, and quantitations were not directly comparable. Therefore, we simply classified cases by the presence or absence of ferruginous bodies on lung tissue digestion for the purposes of this study.

Although the chest-radiograph has traditionally been the imaging technique of choice in screening for asbestosis, high-resolution computerized tomography (HRCT) has emerged as a more sensitive tool in detecting changes consistent with asbestosis [5]. Previous studies have shown that HRCT detected changes suggestive of asbestosis in 80% of patients with clinical but not chest radiographic evidence of the disease [5]. Signs suggestive of asbestosis on HRCT include evidence of interstitial fibrosis (honeycombing and thickening of the septa and interlobular fissures), evidence of diffuse fibrosis (pleural thickening, parenchymal bands and rounded atelectasis), and pleural plaques [1]. Classification schemes for HRCT similar to the ILO classification system used with chest radiography have been proposed, but as of yet, none have been widely accepted [1]. The association of changes found on HRCT and the histologic diagnosis of asbestosis may be a future avenue for research in this area.

As a retrospective review, our study was limited by the nature of the clinical information provided prior to postmortem examination. In many cases, the only clinical information provided was the age, sex, cause of death and presence or absence of an antemortem clinical diagnosis of asbestosis by chest radiography. We were unaware of the patient's chest radiograph ILO classification, so any correlation of autopsy findings with ILO classification was impossible. Because the control group included both cases in which there were "negative" premortem chest radiographs and cases in which there were no premortem chest radiographs, sensitivity and specificity of chest radiography cannot be inferred from this study. More research is needed to compare the antemortem ILO classification and the subsequent findings at autopsy. This may help establish a level of abnormality that would be sufficient to refine the diagnosis of asbestosis.

## Conclusion

This study indicates that an antemortem chest radiograph consistent with asbestosis combined with a history of exposure to asbestos is more predictive of histologically-proven asbestosis at autopsy than exposure history alone. Further studies are needed to evaluate correlation between antemortem ILO classification of chest radiographs and subsequent findings at autopsy.

## Competing interests

The authors declare that they have no competing interests.

## Authors' contributions

EC conceived of the study and gathered the data. KM drafted the manuscript. CM performed the statistical analysis. All authors read and approved the final manuscript.
